# The feasibility of collecting information from people with Multiple Sclerosis for the UK MS Register via a web portal: characterising a cohort of people with MS

**DOI:** 10.1186/1472-6947-12-73

**Published:** 2012-07-18

**Authors:** David V Ford, Kerina H Jones, Rod M Middleton, Hazel Lockhart-Jones, Inocencio DC Maramba, Gareth J Noble, Lisa A Osborne, Ronan A Lyons

**Affiliations:** 1 College of Medicine, Swansea University, Swansea, SA2 8PP, , Wales, UK; 2 Long Term & Chronic Conditions Centre, College of Human and Health Sciences, Swansea University, Swansea, SA2 8PP, , Wales, UK

**Keywords:** Multiple Sclerosis, Disease register, Data linkage

## Abstract

**Background:**

A UK Register of people with Multiple Sclerosis has been developed to address the need for an increased knowledge-base about MS. The Register is being populated via: a web-based portal; NHS neurology clinical systems; and administrative data sources. The data are de-identified and linked at the individual level. At the outset, it was not known whether people with MS would wish to participate in the UK MS Register by personally contributing their data to the Register via a web-based system. Therefore, the research aim of this work was to build an internet-mounted recruitment and consenting technology for people with Multiple Sclerosis, and to assess its feasibility as a questionnaire delivery platform to contribute data to the UK MS Register, by determining whether the information provided could be used to describe a cohort of people with MS.

**Methods:**

The web portal was developed using VB.net and JQuery with a Microsoft SQL 2008 database. UK adults with MS can self-register and enter data about themselves by completing validated questionnaires. Descriptive statistics were used to characterise the respondents.

**Results:**

The web portal was launched in May 2011, and in first three months 7,279 individuals registered on the portal. The ratio of men to women was 1:2.4 (n = 5,899), the mean self-reported age at first symptoms was 33.8 (SD 10.5) years, and at diagnosis 39.6 (SD 10.3) years (n = 4,401). The reported types of MS were: 15% primary progressive, 63% relapsing-remitting, 8% secondary progressive, and 14% unknown (n = 5,400). These characteristics are similar to those of the prevalent MS population. Employment rates, sickness/disability rates, ethnicity and educational qualifications were compared with the general UK population. Information about the respondents’ experience of early symptoms and the process of diagnosis, plus living arrangements are also reported.

**Conclusions:**

These initial findings from the MS Register portal demonstrate the feasibility of collecting data about people with MS via a web platform, and show that sufficient information can be gathered to characterise a cohort of people with MS. The innovative design of the UK MS register, bringing together three disparate sources of data, is creating a rich resource for research into this condition.

## Background

Multiple Sclerosis (MS) is a degenerative neurological disorder that is estimated to affect around 85,000 to 100,000 people in the UK [1,2]. Programmes for treating and managing MS should be underpinned by robust data, but it has been recognised that there is a significant lack of reliable, evidence-based information to inform service delivery and to generate important health service research on MS in the UK [3]. This may be due to a number of factors such as the prolonged disease course, evolving over several decades [4], and difficulties or delays in diagnosis, as people may present with a wide variety of symptoms [5]. To date, there is no central repository for MS data in the UK. There are a number of different database systems in MS treatment centres, but no agreed data model and no network and policy infrastructure for sharing data between NHS settings [3,6]. In response to this, the MS Society of Great Britain and Northern Ireland [2] commissioned the development of the UK MS Register.

The aim of the work was to build a working prototype MS Register, for use in 5 centres across the UK, capable of being scaled to a national deployment. There are two clinical sites in England, one in Wales, one in Scotland and one in Northern Ireland. The Register has been designed to capture three main categories of data and to be able to anonymously link these data at the individual level whilst retaining privacy. Data are collected directly from people with MS via a purpose-built web portal, from patient-management systems operating in NHS neurology clinics, and from sources of routine administrative data (such as primary care and hospital data). Numerous datasets may be included in each of the three categories. There are other national registers for people with MS such as in Denmark, Norway, Sweden, Italy, Germany and North America [7]. However, these collect data from only one or two of these categories, though this may include multiple datasets. By uniting datasets from three disparate categories the UK MS Register model is innovative in its design and provides new opportunities for studying MS via linked data. The Register is operating initially a prevalence-based register, but as we continue to collect data, with regular updates from the different sources, we will be able to estimate incidence. Our aim is to create a UK population Register of people with MS, with as complete coverage as can be obtained whilst acknowledging that participation is voluntary. This register model could also be applied in other disease conditions.

### Research aim

At the outset of this work it was not known whether people with MS would wish to participate in the UK MS Register by personally contributing their data to the Register via a web-based system. Therefore, the objective of the work described here was to build an internet-mounted recruitment and consenting technology for people with MS, and to assess its feasibility as a questionnaire delivery platform by determining whether the information provided could be used to describe a cohort of people with MS. In order to do this, we provide a description of the development of the portal and the self-reported characteristics of its first cohort of people with MS.

## Methods

### Research ethics and governance

The UK MS Register study was granted ethical approval by the South West – Central Bristol Research Ethics Committee (11/SW/0160) under the category of a research database [8]. The Register has been based on the proven technologies and robust Information Governance arrangements in place in the Secure Anonymised Information Linkage (SAIL) system developed by the Health Information Research Unit (HIRU) [9,10]. HIRU aims to realise the potential of electronically held, person based, routinely collected information and to bring together expertise in health informatics to support and conduct research. The SAIL databank holds multiple, disparate person-level datasets drawn from operational and national systems, and over 2 billion anonymous records have been loaded to date. Novel anonymisation, encryption and linkage processes have been developed to manage and link datasets securely, so that the SAIL databank is an extremely successful method of accessing linked data from individuals while preserving their anonymity. Under the ethical approval that has been obtained, data collected via the portal, the neurology clinics and routine administrative sources can be anonymously linked using the SAIL methodologies provided that agreement to the portal terms of service (via the portal) and written informed participant consent (at the clinics) have been obtained. The consent process at the neurology clinics is for the provision of identifiable data to the Register, so that the identifiable details can used to enable record linkage. Datasets are linked at the individual level using first name, surname, date of birth, gender and postcode as the matching variables, and then a unique anonymous identifier is assigned to each individual in the dataset to act as a linking field across datasets. This, together with a de-duplication process, ensures that each person is included on the Register only once. The working UK MS Register contains only anonymous data but facilities are in place to re-contact participants to take part in further research.

### Development and implementation of the web portal

Although the development of the portal is embedded in the SAIL system, it is not specific to SAIL. It uses commonly used toolsets and other systems could equally form the basis of such a portal, either as part of a register or standalone. The portal was developed in Visual Studio 2010, deployed on Microsoft IIS (Internet Information Services) and the data are stored securely in Microsoft SQL Server 2008. The interface elements of the portal are a mixture of HTML and JQuery which is a Javascript library. This mix of technology was used to allow a deeper level of interaction and feedback to the portal users. There are three routes by which people with MS can access this questionnaire-delivery platform: via the standalone register web portal [11], through the MS Society website (available to members of the Society [2], or via a purpose-built web application on Facebook [12]. These entry options are designed to boost recruitment and are ultimately all routed to the questionnaire-delivery platform. People who use the main Register web portal are directly authenticated via the portal. Users select their own usernames, which must be unique, and passwords are stored as salted hash elements within the database, providing a high level of security. The second method makes use of an authentication token from the UK MS Society website. In this case, no identifiable details are transferred between the MS Society and the Register portal, just the token. This allows members of the Society to join the Register without having to remember an additional user name and password. Once logged in and registered, an XML message is returned to the MS Society member. This is displayed to them as an interactive element that allows them to return to the Register at will whilst logged in to the MS Society site. The final method of authentication is via the Facebook social networking site. Web developers within Swansea University created a Facebook application via the published guidelines [13]. Again, only authentication information is provided and no personal or clinical information is shared with Facebook. Users must be logged into Facebook and agree to install the Register application. They then access the Register from the link that the application installs in their Facebook profile.

Following the development process a demonstration portal was implemented for usability testing and improvement. Feedback on its utility was obtained from people with MS and key stakeholders, including clinicians and representatives of the MS Society before the portal was formally launched to the public. The work on usability testing is the subject of another article which is in preparation. The launch of the portal was marked by a nationwide advertising campaign by the MS Society and mailings to MS Society members, radio and TV broadcasts and national newspaper articles [14,15]. People (over 18 yrs) with a diagnosis of MS and living anywhere in the UK are eligible to register on the web portal. Participants must have a functional email address and to be able to agree to the terms of service.

### Data collection and analysis

The web portal collects demographic data from people with MS and hosts a number of validated questionnaires. These instruments were chosen following a literature review and discussions with key stakeholders, including neurology clinicians, people with MS, researchers and the MS Society. They include the Hospital Anxiety and Depression scale [16], the MS Disease Impact Scale-29 [17], and the EQ-5D [18], covering a range of topics such as: MS and mental well-being; MS and quality of life; MS and lifestyle; and medication records. The questionnaire entitled ‘You, your MS and lifestyle’ collects baseline information about MS including: date of diagnosis of MS, type of MS and age at onset, plus information on education, employment and living arrangements for people with MS. Recruitment to the web portal is on-going, and people who register are sent an email every 3 months inviting them to return to the portal and update their questionnaires to build up a longitudinal data source.

The responses to ‘You, your MS and lifestyle’ (obtained between May and July 2011) were used as the sample for data analysis in this work. They were collated with the basic demographic characteristics (such as age and gender) required to register on the portal in order to be able to explore various factors about living with MS. Following quality assurance, the resulting anonymised dataset was analysed (in SPSS v.16) using descriptive statistics to show the sort of information that can be collected and to provide a general profile of the first cohort in the UK MS Register. The characteristics of the respondents were compared with published reports on the prevalent MS population, and employment profiles, ethnic composition, and highest educational attainments of people with MS were compared with the general UK population [19-21].

## Results

The web portal was successfully launched in May 2011 and within three months 7,279 individuals had enrolled on the MS Register via the web portal. Taking into account that there are estimated to be between 85,000 and 100,000 people with MS in the UK [1,2], this represents an approximately 8% sample of the prevalent MS population. Of these respondents, 97% entered directly via the stand-alone website, 2% registered by way of the UK MS Society website, and 1% registered through the use of the Facebook application. Once registered, people can choose whether to provide additional information via completion of the questionnaires. This work relates to ‘You, your MS and lifestyle’ and majority went on to complete this questionnaire. As this questionnaire contains disparate questions, and is not an assessment tool that produces an overall score, it is not necessary for it to be completed it in its entirety in order to be able to use the data. The rate of responses ranged from 61% (UK country of origin) to 82% (types of first symptoms) and numbers of responses are shown in the text with the relevant results.

### Demographics

There were 3,290 respondents who stated they lived in England, 619 in Scotland, 342 in Wales and 177 were of unknown location (n = 4,428). The ratio of male to female respondents was 1: 2.4 (n = 5,899) and there was a broad age range among the respondents at time of enrolment: 18 to 95 years, with a mean age of 50.8 (SD 11.4) years and a median of 50.0 years. The ethnic composition of the group (n = 5,780) was largely White British (93.8%) with a variety of other ethnicities making up the remainder.

### MS and diagnosis

**Figure 1:**
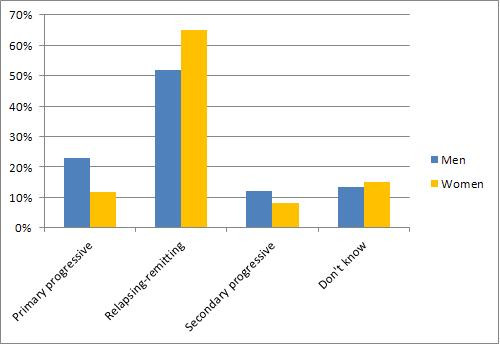
**The distribution of types of MS by gender.** People with MS provided information on the type of MS they have been diagnosed with. The proportions are indicated here, grouped by gender.

MS is classified into different types depending on the characteristics of the disease course. The majority of patients are initially diagnosed with relapsing-remitting MS (RRMS), but this may change over time [2]. Of the respondents that provided their information on this (n = 5,400), 15% reported having primary progressive MS (PPMS), 63% stated that they had RRMS, and 8% had secondary progressive MS (SPMS). The remaining 14% did not know which type of MS they had. The distribution of reported type of MS by gender is shown in Figure 1. It can be seen that relatively greater proportions of men report that they have a progressive type and relatively greater proportions of women report having RRMS. A first-degree family member (parent, sibling or child) with MS was reported by 8.5% of the respondents. Of these 4% had a parent who had MS, 4% had a sibling with MS, and 0.5% stated that they had a child who developed MS.

**Figure 2:**
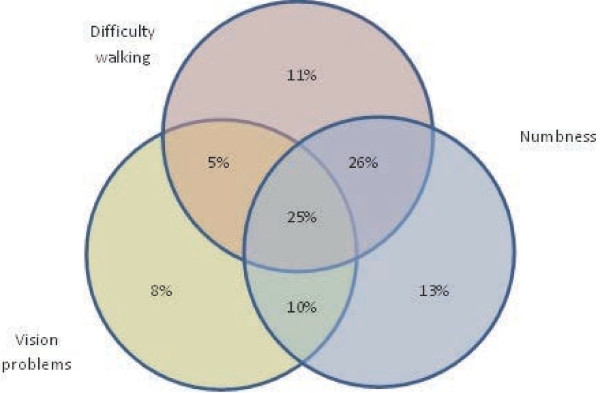
**Initial symptoms experienced by people with MS.** People with MS were asked to indicate whether they experienced numbness, difficulty walking or problems with their vision, or combinations of these symptoms, among their initial symptoms of MS.

The mean age at which the respondents recalled first experiencing symptoms of MS was 34.0 (SD 10.5) years (n = 4,918), and the mean age at which their diagnosis was confirmed was 39.4 (SD 10.1) (n = 4,883). The mean time since their diagnosis was confirmed was 11.4 (SD 8.9) years with a range of 0 to 63 years. This indicates that our sample contains a breadth of respondents spanning recently-diagnosed and long-term. Although there is no definitive list of early MS symptoms, the ones experienced by the respondents (n = 5,969) were among the most common: [2] 67% had difficulty walking, 65% experienced numbness, and 59% had vision problems. These symptoms were sometimes present in combinations: for example, 25% of respondents reported experiencing all three effects and only 2% reported that they did not experience any of these symptoms. A Venn diagram showing the proportions of people with MS experiencing the various combinations of these symptoms is shown in Figure 2.

People with MS reported different experiences in the process of their diagnosis. However, in accordance with the McDonald criteria [22,23], the majority received an MRI scan (79%) and in 29% of cases this was used in conjunction with CSF (lumbar puncture) and clinical findings. Less than 4% (3.7%) did not recall receiving either of these and stated that they were diagnosed from clinical findings alone. Most people (79%) were given the diagnosis of MS by a consultant neurologist, 19% by a consultant physician and less than 2% by a general practitioner (n = 5,736).

### Education and employment

**Figure 3:**
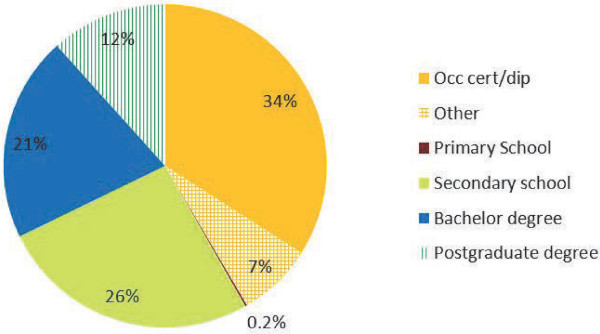
**Highest educational attainment reported by people with MS.** People with MS provided information about their highest educational attainments.

The respondents were asked about their highest educational achievements. Among the 5,819 respondents, the largest single qualification was an occupational certificate or diploma (34%), a third (33%) attained a university bachelor or postgraduate degree, just over a quarter (26%) were educated to secondary school level and only 0.2% left school after primary school. The remainder (7%) indicated that they had miscellaneous qualifications or overseas education (Figure 3).

The people with MS were asked about their usual occupation and the resulting profile (n = 5,046) was compared with the actual employment profile of the general UK population using the official labour market categories for 2010–2011 (Table 1) [19]. It can be seen that in some categories the respondents usual occupations are similar to the actual figures for the UK, and in others, they differ. It should be noted that this compares usual with actual occupations, and our sample consists of approximately 30% men and 70% women, whereas the UK workforce is approximately 54% men and 46% women [19].

**Table 1:** Usual employment types among people with MS

**Employment type**	**Respondents %**	**UK %**
Administrative & secretarial	22.8	10.7
Associate professional & technical	10.4	14.7
Caring, leisure & other personal service	3.9	8.8
Elementary occupations	1.9	11.3
Managerial, director or senior official	15.6	15.6
Process plant/machine operatives	1.2	6.6
Professional	30.5	14.1
Sales & customer service	7.2	7.4
Skilled/trade	6.4	10.3

People with MS provided information on their usual type of occupation. This is compared with the actual employment profile for the UK population (2010–2011). These profiles are discussed in the text.

The responses on actual employment status showed that 36.9% of the sample were in employment, which equates to 41.9% of the people of working age, and of these, the majority were in part-time work (88%). By comparison, 76.9% of the general UK population (aged 16 to 64 years) are economically active [19]. Almost a third of the respondents (29.8%) reported that they were sick/disabled, equating to 32.7% of working age, with the majority reporting that they are permanently (rather than temporarily) sick/disabled (91%). This is higher than the 24.5% of the working-age UK population recorded as being sick/disabled [19]. The differences in the employment and sick/disabled rates between people with MS and the general UK population show the impact of MS on the UK workforce. Over a fifth of the respondents (22.4%) reported that they had retired, and this equates to 86% of the over 65 s age group. The remainder of the respondents were engaged in activities such as education, home-making or voluntary work, or they gave their status as unemployed.

The distributions of the types of MS among the people who were employed were compared to those of people who were sick/disabled. Among the employed respondents 20.3% reported having PPMS, 40.4% RRMS, 10.0% SPMS and 29.3% did not know which type of MS they had. This indicates that, as might be expected, higher proportions of people with RRMS are in work compared to the progressive types of MS. Among the sick/disabled respondents there were 27.6% with PPMS, 20.9% with RRMS, 31.9% with SPMS and 19.6% did not know their type of MS. This is the converse, as it shows that greater proportions of people with a progressive type of MS are sick/disabled compared to people with RRMS.

**Figure 4:**
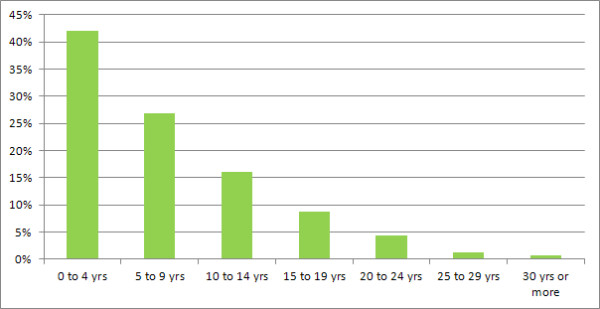
**Employment rates by time since diagnosis.** Proportional rates of employment for people with MS of working age were assessed against the duration of the MS diagnosis in 5 year bands.

**Figure 5:**
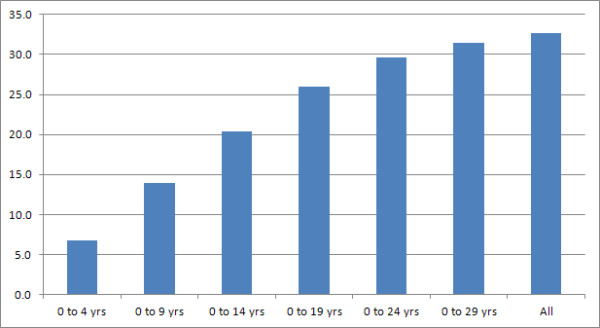
**Rates of sickness/disability status by time since diagnosis.** This shows the cumulative percentage of people with MS of working age reporting their status as sick/disabled by length of diagnosis (in 5 year bands).

The employment rate among the respondents who reported that they were working (<=64 for men and < =59 for women) was assessed against the length of time since their MS was diagnosed (in 5 year bands). As would be expected, this showed that the proportions of people in employment declines with increasing time since diagnosis (Figure 4). For example, over 40% of the people who are working have a recent diagnosis (0 to 4 years), and only approximately 2% of the people who are working have been diagnosed for 25 years or more. A similar analysis was conducted to assess the rate of sick/disabled respondents of working age and, as would be expected, this showed a steady increase in cumulative proportions by time since diagnosis (Figure 5).

### Household arrangements

There were a wide range of living arrangements among the respondents (n = 5,604). The most frequently reported household settings for the people with MS were as: an adult couple not on a pension and with (non-dependent and/or dependent) children (25%); an adult couple on a pension with no dependent children (23%); an adult couple not on a pension and with no children (16%); and a single pensioner (15%). Some respondents in adult couples reported that they live in households with other relatives or unrelated adults (8%). Altogether there were 31% with dependent children and 3.3% were single parents with dependent children.

## Discussion

### Main findings of this study

The main findings of this study are two-fold. Firstly, we have shown that it is possible to collect information from thousands of people with MS via a web-based questionnaire delivery platform. Secondly, sufficient information can be gathered to characterise a cohort of people with MS to feed into the Register. This provides new information about the lives of people with MS in the UK from their own perspective. This is encouraging as it supports the continuance of collecting information for the UK MS Register via this route, and this design could be used to set up a similar portal to collect data from people with other conditions.

### What is already known on this topic

There are numerous research programmes seeking to understand the possible causes of MS and to develop drug therapies to manage the condition. However, it is acknowledged that there is a great shortage of information to guide service delivery and research about MS [3], and this led the MS Society to commission a UK MS Register. It is estimated that (in 2008) there were approximately 85,000 people with MS in the UK, with a mean age of onset of 32.5 years, and gender proportions of 1:2 to 1:3 male to female [1,2].

### Data validity

Through the analyses we have conducted we have shown that we have a broad sample of ages and MS durations and that our respondents are reasonably representative of the prevalent MS population. The gender proportions, mean age at onset, proportions of MS types, and the distribution of types of MS by gender in our findings are similar to other reports [1,2,24]. We cannot yet estimate prevalence from this initial, unlinked dataset, but we will be able to do this in the near future when we are able to link portal responses with other sources of data.

We compared various features of the sample with the general UK population. In the 2001 census, approximately 85.7% of the UK population reported that they were White British, and 91.4% White British/Irish/Other, with the remainder being made up of various ethnic groups [20]. There may be minor changes in the UK population over time since the census was taken but the higher proportion observed in our sample (93.8%) is not unexpected as higher rates of MS are reported in White populations [25]. The respondents were similar to the UK population in highest educational attainment. The UK Labour Force survey for 2011 showed that 31% held a first degree or a higher degree, and 29.3% did not progress beyond secondary education [19]. In our sample 33% held a first degree or a higher degree, and 26.2% were educated to secondary level. People with MS were asked about their usual occupations and these were compared with the actual occupations of the UK workforce, and although there were some similarities, some obvious differences were seen in the occupation profiles (Table 1). The full reasons for these differences are not known at this stage. However, it is worth noting that the comparison was of usual occupations with actual occupations, and it is possible that differences would also be noted if the usual and actual occupations of the general UK population were compared, as people may work in jobs outside their usual occupation type. Only approximately 42% of our potential MS workforce were in employment and it is possible that the observed profile also reflects the types of occupation that people with MS are able to continue working in. Also, the gender proportions of our sample are different to those of the UK workforce, and this may influence the distribution of occupation types with some types of work being more common to one gender or the other. Although further work needs to be carried out to understand the occupation profiles, the analyses on employment status, namely, the distributions of types of MS among people who are working and among people who report that they are sick/disabled, and the trends in employment rates and sickness/disability rates over time are in keeping with what would be expected in a degenerative condition like MS.

### What this study adds

In collecting data from clinical settings, administrative datasets and directly from people with MS, the UK MS Register has a broader data model than other national MS Registers which use only one or two categories of data [7,26]. This model could be applied to other disease Registers. The web portal provides a flexible platform for the delivery of a range of questionnaires, providing all people with MS in the UK an opportunity to contribute their data, which is of potentially great value to service planning and research. This study has provided information from people with MS on their demographics, the process of their diagnosis and the type of MS they believe they have, and on their education, employment and living arrangements.

### Future work

Future work will include analysis of the responses to other questionnaires delivered via the platform, to create a fuller picture of living with MS. We will also link and compare the self-reported information with clinical and administrative data to estimate disease prevalence and to carry out further validation. Since MS can be difficult and lengthy to diagnose, we are developing a case ascertainment algorithm to use against routine data (such as primary and secondary care records). This will help us to study the anonymous routine data to profile the group of people with MS who are not on the Register and thus enable us to target further recruitment activities. We are engaging in a series of marketing activities to promote the Register and to encourage further participation, and through these methods we are seeking to ensure that the Register is inclusive and representative of the UK MS population. Furthermore, a programme of qualitative research is underway to engage with people with MS to improve our understanding of their needs in contributing to the Register. For example, we are seeking to understand their internet preferences, such as their reasons for choosing a particular portal entry route, so that we can optimise enrolment on the Register [27]. In future, the Register data will be made accessible for analysis, subject to regulatory and governance requirements. The final operating model for these arrangements is yet to be determined.

### Limitations of this study

The information used in this study was self-reported and the respondents are not necessarily a fully representative sample of people with MS in the UK. Ascertainment is commonly assumed to be skewed when using web-based methods, as the technology may pose a barrier to the elderly, disadvantaged, technically inexperienced or cognitively impaired [28,29]. Response bias in portal data will be addressable using the linkable data from clinical sites and routine sources, as the Register continues to build up an increasingly detailed picture of MS in the UK and to become a rich information resource.

## Conclusions

The initial findings from the MS Register portal demonstrate the feasibility of collecting data about people with MS via a web-based questionnaire delivery platform, and show that sufficient information can be gathered to characterise a cohort of people with MS. The innovative design of the UK MS register, bringing together three disparate sources of data, is creating a rich resource for research into this condition.

### Funder

The development of a UK MS Register was commissioned by, and is supported by a grant from, the MS Society of Great Britain and Northern Ireland.

## Competing interests

The authors declare that they have no competing interests.

## Authors’ contributions

All authors contributed to the design of the project, and RMM, HL-J, KHJ and IDCM acquired, analysed and interpreted the data. KHJ drafted the manuscript and all authors revised it critically for important intellectual content, and gave their final approval of the version to be published.

## Pre-publication history

The pre-publication history for this paper can be accessed here:

http://www.biomedcentral.com/1472-6947/12/73/prepub
